# Young People's Use of Digital Tools to Support Their Mental Health During Covid-19 Restrictions

**DOI:** 10.3389/fdgth.2021.763876

**Published:** 2021-12-01

**Authors:** Claudette Pretorius, David Coyle

**Affiliations:** ^1^Insight Centre for Data Analytics, University College Dublin, Dublin, Ireland; ^2^School of Computer Science, University College Dublin, Dublin, Ireland

**Keywords:** COVID-19, mental health, help-seeking, young people, isolation

## Abstract

Young adulthood represents a sensitive period for young people's mental health. The lockdown restrictions associated with the COVID-19 pandemic have reduced young people's access to traditional sources of mental health support. This exploratory study aimed to investigate the online resources young people were using to support their mental health during the first lockdown period in Ireland. It made use of an anonymous online survey targeted at young people aged 18–25. Participants were recruited using ads on social media including Facebook, Twitter, Instagram, and SnapChat. A total of 393 respondents completed the survey. Many of the respondents indicated that they were using social media (51.4%, 202/393) and mental health apps (32.6%, 128/393) as sources of support. Fewer were making use of formal online resources such as charities (26%, 102/393) or professional counseling services (13.2%, 52/393). Different social media platforms were used for different purposes; Facebook was used for support groups whilst Instagram was used to engage with influencers who focused on mental health issues. Google search, recommendations from peers and prior knowledge of services played a role in how resources were located. Findings from this survey indicate that digital technologies and online resources have an important role to play in supporting young people's mental health. The COVID-19 pandemic has highlighted these digital tool's potential as well as how they can be improved to better meet young people's needs.

## Introduction

The COVID-19 outbreak has impacted day-to-day living worldwide; from early 2019 many countries across the world implemented periodic “lockdown” measures, requiring households to stay at home and isolate in order to curb the spread of the virus (1). This involved the closing of schools and colleges, as well as pausing various support services, including counseling and other mental health services (2). Whilst these measures were necessary, their effect on individual's mental health and well-being is worrying (3).

Prior to the onset of the pandemic, the prevalence of mental ill-health and mental illnesses amongst young people was already an area of worldwide concern, with a reported 25% of young people aged between 12 and 25 experiencing a mental illness (4–7). A young person's mental health, educational achievement and social relationships are impacted by the availability of personal and contextual resources that can support them through this period (8). The consequences of young people not receiving the help they need can be significant, including developing chronic mental health conditions, lower educational attainment and poor interpersonal relationships (9, 10).

The impact of COVID-19 specifically on the mental health of young people is already evident, with young people being more likely to experience mental distress as compared to other population groups (11, 12). Numerous studies report an increase in the presentation of symptoms of mental ill-health, including acute stress, anxiety and depression (2, 13–16). A rapid review conducted by Millar et al. (16) indicates that young people experienced increased levels of distress, anxiety and depressive symptoms during the COVID-19 pandemic; with many between the ages of 16 and 25 reporting high levels of ‘lockdown loneliness'. Furthermore, a longitudinal study by Cresswell et al. (13) has found that children and young people experienced increased behavioral and attentional difficulties during peak periods of restrictions. In Ireland for example, where the research in this paper was conducted, a study by O'Sullivan et al. (17) indicated that parents of both children and adolescents reported that their children's mental health had negatively been impacted by lockdown measures, with many struggling with isolation, loneliness, and feelings of anxiety.

Help-seeking has been identified as an important protective factor to young people's mental health (18). Failing to seek help has been associated with negative health outcomes, including substance abuse, self-harm, poor quality of life and premature death (19). Ougrin (20) reports that there was a substantial decrease in the number of presentations to hospital emergency rooms by young people for psychiatric reasons during lockdown. This is concerning as help-seeking plays an important role in self-harm and suicide prevention (21). Under normal circumstances help-seeking is often impeded by factors such stigma, poor mental health literacy and difficulty in accessing services (22). Lockdown measures further contributed to these difficulties in access. Additionally, it is known that young people have preference for informal sources of support including friends, peers, and family (23). Emerging adulthood, the period from late teens to early/mid-twenties, is a period marked by a need for increased interactions with peers, exploration and experimentation (24, 25). Lockdown measures disrupted young people's ability to engage in these behaviors but also their access to offline resources of mental health support (14). Despite increases in depressive and anxiety symptoms, research shows that there were no increases in reported help-seeking however many young reported engaging in self-help behaviors (26). Although some of the usual avenues for support are heavily interrupted during periods of restrictions; young people are using alternative means, including online resources, to support their mental health (12, 14).

Whilst some are cautious about the use of technology and the Internet for online mental health services (27); the accessibility of the Internet has created the opportunity for more resources and sources of help and information to become available to young people (12). Throughout lockdown, it was often suggested that young people make use of technologies such as video chat and other applications to stay connected to peers and family; with digital technologies being recognized as important tools to support mental health during the pandemic (25, 28). Additionally, it was found that increased levels of psychological distress were often associated with increased use of digital tools to support mental health during lockdown periods (29–31).

It is well-established that prior to the pandemic, young people make use of online help-seeking resources to support their mental health (32). Seeking online resources starts as early as the age of ten with some even making use of gaming devices to do so (33). Young people often engage in online help-seeking due to its increased accessibility and privacy. It also satisfies their preference for self-reliance (34). Young people often make use of Google search to find relevant online mental health resources, a process that is either helped or hindered by their level of mental health literacy (35). Previous research has also identified that young people use a multitude of resources to seek help online, including resources from government or charities, mobile applications, and social media (34). The use of social media and its effects on young people's mental health is often an area of debate (36). Concerns have been raised that social media has been linked to increases in depressive and anxiety symptoms but also that it promotes social comparison pressure and in some cases social isolation (37). However, the cross-sectional nature of many of these studies makes it difficult to make causal inferences (38). On the other hand perceived benefits of using social media include the facilitation of social interaction and access to a peer support network (37). Social media use increased during the pandemic (39); with many young people using social media to cope with the effects of lockdown as well as overcome feelings of loneliness and isolation (40, 41). Similarly, there were reported increases in the downloads of various mental health apps during the pandemic (42). Whilst excessive use of digital technology and its impact during the pandemic remains a concern, little is known about young people's specific use of digital tools to support their mental health. An exploratory research design was selected to understand how young people in Ireland supported their mental health during lockdown. A cross-sectional online survey was conducted with 393 young people exploring which online resources they were using to support their mental health during the first lockdown.

## Methods

### Study Design

An online cross-sectional survey approach was selected to assess how young people, living in Ireland, were using technology and Internet-based resources to support their mental health whilst in lockdown during the COVID-19 pandemic. The survey was carried out between 22 April and 22 May 2020 when the number of COVID-19 cases were increasing, and the Irish government had imposed level 5 lockdown restrictions to limit the spread of the virus. Level 5 restrictions required individuals to stay home avoiding contact with others and only leaving their home for essential purposes. Inclusion criteria were (i) being a resident of Ireland and (ii) aged between 18 and 25. Ethics approval for this research was provided by the University College Dublin Office of Research Ethics (LS-20-23-Pretorius-Coyle). All data was collected through an anonymous online survey.

### Survey Procedure

This study used a survey link to direct participants to the survey. Participants were recruited from various social media platforms commonly used by young people (e.g., Facebook, Instagram, Twitter, and SnapChat). The social media advertisements were specifically targeted to appear on the feeds of Irish users between the ages of 18 and 25. The survey was hosted on LimeSurvey on a server based in the European Union (Germany). The first component of the survey consisted of the information page, which included information regarding the purposes of the study, how the data would be used, anonymity, confidentiality, and data protection. Participants were then asked to provide consent and confirm that they were both between the ages of 18–25 years old and living in Ireland, if they wished to continue with the survey. Information on mental health support was provided on the landing page of the survey as well as on the survey termination page. The survey consisted of 15 questions, over 6 screens, and took between 5 and 10 min to complete (see Supplementary Material for survey questions). Multiple responses from the same user were prevented by using cookies, and no incentive was offered. Participants were permitted to skip any question they did not wish to answer. In total, 742 participants began the survey, with a total of 393 successfully finished the entire questionnaire. Data from uncompleted surveys were not used as withdrawal from the survey indicated the withdrawal of consent.

### Survey Measures

The survey consisted of both open and closed response questions to assess: (1) demographics (2) online resources currently being used to support mental health. This question was broken down into subsections focusing on specific online resources including social media, discussion boards, formal services, mobile applications, messaging platforms, and professional services. Additionally, participants were asked which specific accounts/services they followed or used for each of the respective resources. The survey then continued by asking: (3) how online resources were located; (4) which online resources were being used prior to the pandemic; (5) online resources that they started using during the lockdown; (6) which resources they would continue using post-lockdown; (7) factors that make a good online resource; (8) what is missing from current online resources.

### Data Analysis

Only completed surveys were analyzed. Closed response question data was analyzed IBM SPSS Statistics for Mac, using descriptive statistics. Open response questions were analyzed using either inductive content analysis (43) (sections 2, 5, and 6 of the survey) or thematic analysis (44, 45) (sections 7 and 8 of the survey), using Nvivo 12 for Mac.

When conducting the inductive content analysis, the first author (CP) screened the responses and proposed a codebook for categorizing them based on account focus (e.g., body positivity, mental health, or physical fitness). The codebook was then evaluated by the second author (DC) who provided suggestions. Consensus was reached between both authors on the categorizations.

The data from sections 7 and 8 of the survey were entered into NVivo 12. Data was then coded using Braun and Clarke's (44) six phases of thematic analysis. Phase one began with the first author (CP) screening the data to note initial ideas and codes. The second author (DC) assisted with the second phase of the process and initial codes were discussed. A final codebook was then agreed upon by both authors. CP then recoded the entire dataset. Codes were then organized into themes and themes were then reviewed in relation to coded extracts by DC. Themes were agreed upon by both authors.

## Results

### General Characteristics of Participants

A total of 393 participants took part in the study, of which 81.7% (321/393) identified as female, 12.5% (49/393) identified as male, and 4.1% (16/393) identified as non-binary. The mean age of the population was 19.7 (SD 2.07). The survey had good national coverage, with respondents from all Irish counties, and 59.0% (232/393) of the sample were currently living in a city/town whilst 39.4% (155/393) were living in a rural area. Of the whole sample, 41.7% (164/393) reported their current level of education to be 6th year (final year of high school in Ireland) and 36.4% (143/393) reported their current level of education to be at an undergraduate level.

### Online Resources Currently Being Used to Support Mental Health

#### Social Media

**Figure 1:**
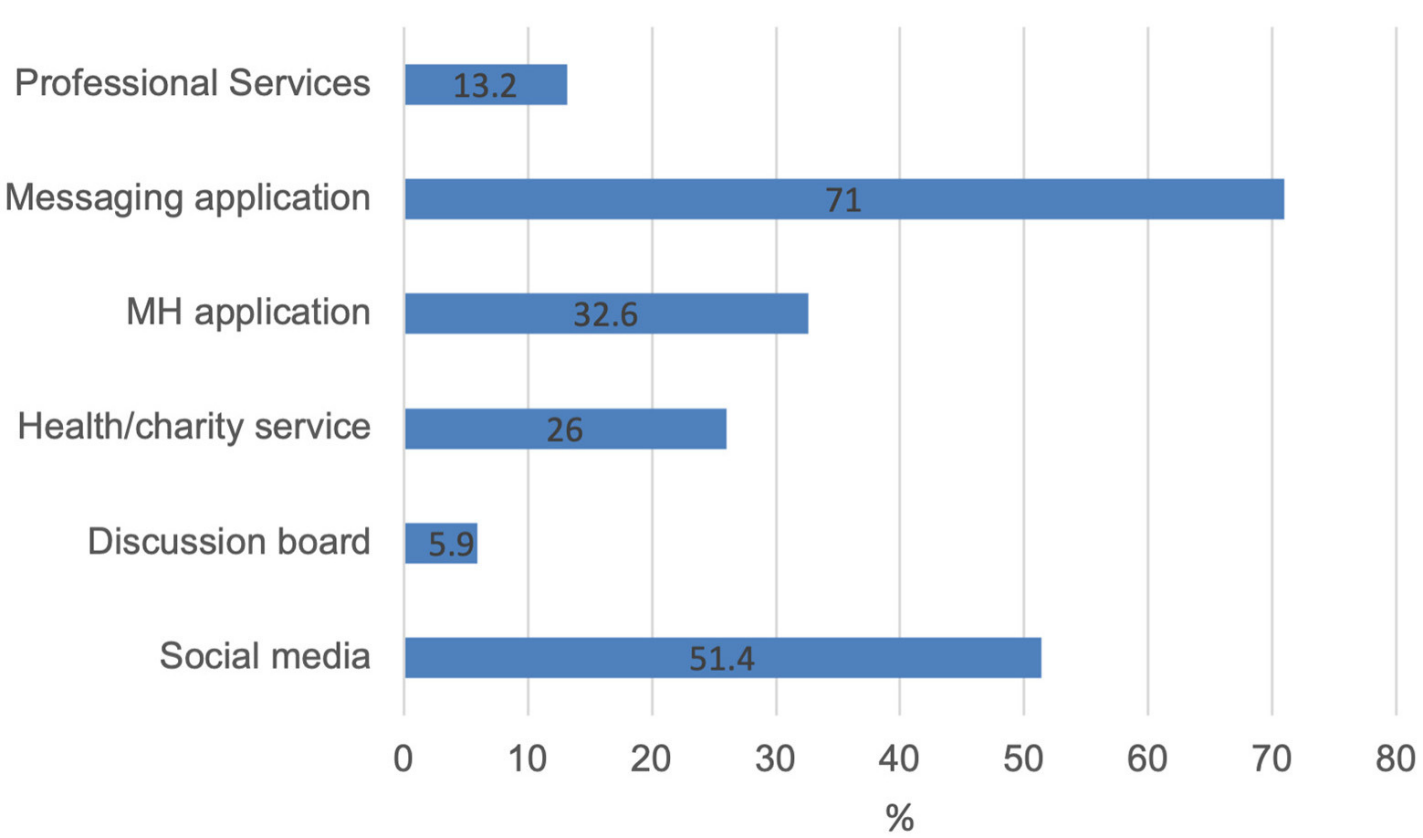
Digital tools used to support mental health.

Of the overall sample, 51.4% (202/393) indicated that they were using social media to support their mental health, see Figure 1. As shown in Table 1 below, Instagram was the most popular form of social media to use, with 38.7% (152/393) of the sample indicating that used it to support their mental health. There was distinct difference in the types of accounts followed across different social media platforms. Instagram was used to follow several influencers, including body positivity influencers (for example @Bodyposipanda); influencers who focused on mental health issues; and influencers focusing on health and fitness. Several participants also indicated following specific mental health accounts such as @hyppyhands. Accounts not related to mental health were also followed including animal accounts, accounts providing inspirational quotes and accounts focusing on art. A similar pattern was found for YouTube, with many participants (*n* = 52) indicating that they followed various influencers, including influencers with a focus on mental health. Several participants also indicated that they used YouTube to access home workouts or that they followed fitness influencers. Facebook use was characterized by the use of support groups; participants indicated accessing support groups focusing on specific issues such as eating disorders or ADHD. Some participants specified that they followed formal support services such Samaritans and other groups focused on mental health such as Mindfulness Ireland. TikTok use to support mental health was characterized by serendipitous finds. That is participants did not access this platform looking for specific content but rather content found randomly that would provide distraction or lift their mood. This tactic was also used on Instagram and YouTube.

**Table 1:** Use of social media platforms.

**Platform**	**Yes**	**No**	**N/A**
Instagram	38.7% (152/393)	12.7% (50/393)	48.6% (191/393)
Facebook	9.2% (36/393)	42.2% (166/393)	48.6% (191/393)
Twitter	4.8% (19/393)	46.6% (183/393)	48.6% (191/393)
TikTok	12.7% (50/393)	38.7% (152/393)	48.6% (191/393)
YouTube	23.7% (93/393)	27.7% (109/393)	48.6% (191/393)
Tumblr	1.8% (7/393)	49.6% (195/393)	48.6% (191/393)
LinkedIn	0% (0/393)	51.4% (202/393)	48.6% (191/393)

#### Discussion Board

Discussion boards were less popular; 5.9% (23/393) of the sample indicated that they were using discussion boards to support their mental health whilst social distancing. Whilst very few participants indicated using discussion boards, they reported following Reddits or boards focusing on depression, confessions, and anxiety.

#### Health or Charity Services

Of the overall sample, 26% (102/393) indicated that they were using a health service or charity website to support their mental health. The most accessed resource was a charity organization called SpunOut (13.2%, 52/393), followed by national health service, the HSE (10.4%, 41/393). Table 2 details the other services used, including the NHS from the UK, BodyWhys and MyMind.ie.

**Table 2:** Use of health or charity services.

**Service**	**Yes**	**No**	**N/A**
HSE	10.4% (41/393)	15.5% (61/393)	74.0% (291/393)
Jigsaw	5.9% (23/393)	20.1% (79/393)	74.0% (291/393)
SpunOut	13.2% (52/393)	12.7% (50/393)	74.0% (291/393)
Samaritans	3.3% (13/393)	22.6% (89/393)	74.0% (291/393)
Pieta house	3.6% (14/393)	22.4% (88/393)	74.0% (291/393)
Turn2Me	2.5% (10/393)	23.4% (92/393)	74.0% (291/393)
A lust for life	1.0% (4/393)	24.9% (98/393)	74.0% (291/393)
Aware	2.3% (9/393)	23.7% (93/393)	74.0% (291/393)
Other	3.6%(14/393)	21.8% (86/393)	74.0% (291/393)

#### Mental Health Mobile Applications

Nearly a third of the sample, 32.6% (128/393), indicated that they were using a mental health app to support their mental health. As shown in Table 3, the most commonly used mobile application was the Headspace app with 16.5% (65/393) of the sample using it. When asked which “Other” mobile applications they were using, participants indicated using “Calm Harm,” “Moodpath,” “#selfcare,” “Woebot,” and “Youper.”

**Table 3:** Use of mental health mobile applications.

**Service**	**Yes**	**No**	**N/A**
Headspace	16.5% (65/393)	16.0% (63/393)	67.4% (265/393)
Calm	7.9% (31/393)	24.7% (97/393)	67.4% (265/393)
Silvercloud	2.5% (10/393)	30.0% (118/393)	67.4% (265/393)
Other	14.0% (55/393)	18.6% (73/393)	67.4% (265/393)

#### Messaging Applications

Messaging applications were most popular, 71.0% (279/393) of respondents indicated that they had used a messaging application to support their mental health during the lockdown. An overview of the applications used in provided in Table 4. When asked whom they contacted using each application, it was apparent that SnapChat was mostly used to contact friends; WhatsApp to contact both family and friends; Messenger to contact friends and less frequently family. WhatsApp and Skype were used as tools to contact a mental health professional for the purposes of therapy or counseling. Other commonly used applications in this category included Zoom and the direct messaging function on Instagram.

**Table 4:** Use of messaging applications.

**App**	**Yes**	**No**	**N/A**
Snapchat	52.7% (207/393)	18.3% (72/393)	29.0% (114/393)
Messenger	34.9% (137/393)	36.1% (142/393)	29.0% (114/393)
WhatsApp	43.8% (172/393)	27.2% (107/393)	29.0% (114/393)
Viber	2.0% (8/393)	69.0% (271/393)	29.0% (114/393)
WeChat	0.0% (0/393)	71.0% (279/393)	29.0% (114/393)
Skype	6.6% (26/393)	64.4% (253/393)	29.0% (114/393)
Telegram	0.5% (2/393)	70.5% (277/393)	29.0% (114/393)
Discord	9.4% (37/393)	61.6% (242/393)	29.0% (114/393)
Other	6.9% (27/393)	64.1% (252/393)	29.0% (114/393)

#### Professional Services

A small percentage of the sample, 13.2% (52/393), indicated that they were using online professional counseling services to support their mental health. Most participants (8.7%, 34/393) were making use of “Other” services not listed in the survey, these services included personal therapist and their college/school mental health services (see Table 5).

**Table 5:** Use of professional services.

**Service**	**Yes**	**No**	**N/A**
MyMind	0.8% (31/393)	12.5% (49/393)	86.8% (341/393)
TherapyHub.ie	0.5% (2/393)	12.7% (50/393)	86.8% (341/393)
CounsellingOnline.ie	0.8% (31/393)	12.5% (49/393)	86.8% (341/393)
BetterHelp	1.3% (5/393)	12.0% (47/393)	86.8% (341/393)
7Cups	1.3% (5/393)	12.0% (47/393)	86.8% (341/393)
Other	8.7% (34/393)	4.5% (18/393)	86.8% (341/393)

#### Locating Mental Health Resources Online

The majority [64.1% (252/393)] of participants indicated that they made use of Google search to locate the online mental health resources they were using to support their mental health. Prior awareness of specific mental health supports (47.1%, 185/393) and recommendations from friends and peers (43.3%, 170/393) also played an important role in locating online resources. Other referral resources included their General Practitioner (GP) and a mental health professional (see Table 6).

**Table 6:** Locating mental health resources online.

**Source**	**Yes**	**No**
Google search	64.1% (252/393)	35.9% (141/393)
Social media ads	29.3% (115/393)	70.7% (278/393)
Recommendations from friends or peers	43.3% (170/393)	56.7% (223/393)
Recommendations from school or college	30.0% (118/393)	70.0% (275/393)
Recommendations from parents	5.9% (23/393)	94.1% (370/393)
Prior awareness of specific mental health supports	47.1% (185/393)	52.9% (208/393)
By following links of websites already used	13.7% (54/393)	86.3% (339/393)
Other	4.1% (16/393)	95.9% (377/393)

### Online Resources Used Prior and During Lockdown and Intentions for the Future

This section explored which resources were already in use prior to the pandemic, which participants started to use because of the pandemic, and which they intended to keep using post-lockdown. An inductive content analysis approach was used and showed that the most used resources pre-lockdown included Headspace, SpunOut, Instagram and other social media. As a result of lockdown, participants indicated that they started to make use of a variety of digital tools including personal therapy online, Headspace, Jigsaw Mental Health services (an Irish charity service), Zoom and social media to facilitate home workouts.

Participants were then asked if they would and why they would continue to use these online resources post-lockdown. Thematic analysis was used to analyze responses to this question. Responses could be grouped into either positive or negative sentiments toward ongoing use of online resources. The following themes were identified as reasons for ongoing use of online resources: “an overall beneficial experience” and addressing an ongoing need. Table 7 in the Supplementary Material provides an overview of these themes with illustrative quotes. The themes “online resources used as a substitute for offline experiences” and “unhelpful experiences,” encapsulate the negative sentiments toward continued use of online resources for mental health support. These themes are discussed in more detail below.

The “overall beneficial experience” theme indicated that many young people had had a helpful experience from the online resources used and for this reason they would continue to use those resources. For some the lockdown created a need to use an online resource that the young person would never have come across or interacted with otherwise, “Yes. I did not realize how helpful spunout really was. I've never given it a chance before. Desperate times made me!”

Respondents indicated that they were pleasantly surprised and experienced a sense of belonging online which reduced feelings of isolation. It was indicated that the information that online resources provided were very helpful and played an important role. Additionally, many resources seemed to promote physical activity as a coping behavior which many found beneficial, “I have found that working out has really uplifted my mood.”

The “ongoing need” theme indicated there would be a need for ongoing mental health support outside of lockdown and for that reason, young people would continue to use online resources. Within this theme it was evident that some young people continued to use mental health resource they were previously using to support their mental prior to the pandemic, “In general my main struggles are to do with isolation & loneliness, so it hasn't really changed since lockdown hence, I've just continued using them.”

This theme also highlighted that many found online resources very accessible and for this reason would continue to use them, “.it's handy to have something that I can access remotely online from the comfort of my own home.”

However, some respondents did indicate that they would not continue to use online resources as they preferred in-person supports, whether this was with a mental health professional or with their peers. Respondents indicated that having the ability to discuss concerns with peers face-to-face was very helpful and that online resources could not replace that experience. “I'll definitely go back to CBT in person–more privacy than at home and I'm not worried about people over hearing me. It does work very well-though.”

The unhelpful experience theme indicated that some respondents did not benefit or receive the help that they required from online resources and for this reason would not continue to use them after the lockdown, “No because it didn't help.” In some cases, this referred to specific resources whilst for others it referred to all of them.

### What Makes a Good Online Resource?

This open response question asked respondents to indicate what they thought would make a good online mental health resource. The following themes were identified: interpersonal connection; content; accessibility and functionality; see Table 8 in the Supplementary Material for further details, including illustrative quotes. The interpersonal connection theme indicated that it was important that an online resource provided an experience where the participant could feel connected to another, whether that be a professional or peer, “Ability to talk to another person (not just articles, videos, etc).” It was deemed helpful if an online resource created opportunities for young people to connect with others either synchronously or asynchronously either though discussion forums or online chat options. It was also important that young people had an empathic experience through these interpersonal interactions, that they felt heard and understood as opposed to receiving generic responses or being sign-posted somewhere else, “Ability to disclose all information and feel listened to and heard rather than just receiving a common and quick remedy.”

The content theme revealed that there was a preference for varied content on online resources including easy-to-read information, personal stories, links to other resources as well as activities and skills-building exercises. Additionally, this theme indicated that respondents would like to see features on online resources that allow them to interact with the content through polls, comment sections and quizzes, “Interactive options–polls, comments, short reminders that the resource is for the user.”

Accessibility was highlighted as an important consideration in the design of online resources. Accessibility was understood not only in terms making resources accessible for those with visual or physical impairments but also in terms of the language used. Anonymity and confidentiality were also important considerations in this theme. It was also indicated that paywalls were seen as significant barriers and currently impede access to some online mental health resources, “Accessible (free is best, not in the position to pay for therapy/counseling).”

The functionality theme indicated which features need to be incorporated into an online resource. Respondents indicated that different options of communication should be made available through the resource including chat, discussion boards and comments sections. It was also highlighted that resource should offer different tailoring options so that help-seekers could easily find relevant information and content within the resource. Finally, good, clear design was emphasized as important as it helped help-seekers feel less overwhelmed when navigating the resource, “Simple, clear layout. Chances are someone visiting the resource may already be overwhelmed.”

### What's Currently Missing From Online Resources

This was the final question in the survey, and asked respondents what they thought was missing from the online resources they were currently using to support their mental health. The following themes were identified: accessibility; interpersonal connection; tailoring; meaningful, relevant choices; visibility; and design and layout; see Table 9 in the Supplementary Material for further details and illustrative quotes.

The accessibility theme refers to various barriers young people encountered when accessing online resources that hindered access or engagement with these resources. Participants indicated that online resources need to provide content and services for young people from under-represented or marginalized groups, with some respondents referring to resources specifically related to LGBT+ issues. It also indicated that respondents were looking for resources that took a less pathologized approach or did not only address those with mental illnesses but rather acknowledged periods of poor mental health. “I find some resources present information from a pathological standpoint e.g., mental health disorders but often all I need is advice for having a few bad days or a stressful few weeks.” Finally, access to online resources were often impeded by payment barriers. For many respondents it was not feasible to access certain features within a resource because of paywalls.

The interpersonal connection theme highlighted the importance of feeling connected to others whilst accessing an online resource. This could be through direct contact facilitated by chat or indirect contact through personal stories or discussion boards, “Discussion boards perhaps would be helpful where we could post an anonymous question and discuss it with other young people.” Respondents also indicated that contact with both professionals and peers was of importance to them and this was lacking from many resources.

The tailoring theme speaks to the extent that an online resource provided personalized content and services. Respondents expressed a need for guidance and activities that were relevant to their needs and situations as opposed to the generic advice they were currently receiving from online resources, “A lot of advice is very generic and doesn't fit a lot of people. We need to hear from a range of different people to dinf something instead of just hearing generalized tips and tricks.”

The meaningful, relevant choices theme highlights the need for online resources to provide varied options in terms of the content, information, and activities. Participants indicated frustration with information that was “dumbed down” or that did not speak to the experiences of young people, “More indepth information for someone like me who wants to understand more about why and how I'm feeling. Just because we're young doesn't mean the information can't be detailed. Also, resources addressing what we're actually going through.” Furthermore, they wanted varied content and formats that would meet different preferences and ways of digesting information.

Respondents commented on the difficulty in finding appropriate online resources and that online resources should be more the visible, “Current resources seem to be passively giving information and allowing people to find it instead of playing an active role in putting forward their information and actively helping you as an individual as opposed to a generalized group of people with all different issues.” The visibility theme addresses this difficulty. Participants mentioned that online resources should do more to be found by advertising on specific issues on other platforms with more user traffic.

Finally, design and layout were again noted by respondents. They wanted design that made them feel comfortable and that the resource was relatable, “A friendly user interface that looks inviting and allows you to feel like asking for help is totally fine.”

## Discussion

The results of this study provide evidence that young people used a multitude of online resources to support their mental health during the first lockdown. Findings suggest the use of both formal and informal online resources, with many discovering new sources of support because of the lockdown measures. This supports other research that suggests that young people engaged in various self-help behaviors, including the use of a variety of digital tools, to support their mental health throughout the pandemic (26, 29, 31). Given the impact of loneliness on young people's mental health during the COVID-19 pandemic, it is important that researchers and service providers understand how online resources can be used to best meet young people's needs to support their mental health.

A quarter (26%, 102/393) of respondents reported using formal services from charities or health services. Given that these services are free these numbers seem low, but this is likely due to a lack of awareness that these resources are available. It appears that for some of the respondents this was the first time they had had the need to use online mental health supports and it is evident that they were pleasantly surprised by some of these services once they discovered them and would continue to use them after lockdown. Poor mental health literacy and lack of awareness of available resources has previously been recognized as a barrier to online help-seeking and likely also played a role during this period (32, 34). The use of formal counseling services was also low, and this is likely due to the fact that few of these services are free. Paywalls have previously been identified as barriers to online help-seeking (32). It seems that some of those using formal counseling services had been using these services prior to the pandemic and migrated with these services online once lockdown was implemented. Future research could investigate whether the use of formal counseling services online increased as the pandemic persisted.

The introduction of lockdown measures appears to have initiated an increased interest and adoption of mental health apps at an overall population level; a study by Wang and colleagues (42) noted an overall increase in the number of downloads as and use of 16 popular mental health apps, including Calm and 7Cups. A study in the United States found that a large proportion (69%) of the young people they surveyed reported using mhealth apps (31). The findings from this survey reflect lower reported app usage, with a third (32.6%, 128/393) of participants making use of a mental health app to support their mental health. It has been well-established that young people have a preference for self-reliance when experiencing mental health concerns (21, 34). It would be worth investigating in the future whether the use of mental health apps satisfies this need for self-reliance for young people or is it other factors such as privacy and cost that influence their use.

Many respondents (51.4%, 202/393) indicated that they used various social media platforms to support their mental health during the initial lockdown. A study by Cauberghe et al. (40) also found that young people used social media as a means cope with the current situation as well as to maintain social contact with peers and family. The results also show that different social media platforms were used for different purposes and different coping strategies. It appears that whilst some platforms such as Instagram were used to follow specific influencers or specific mental health hashtags for advice and guidance; others such as Facebook were used to engage in support groups; and platforms such as YouTube and TikTok were sometimes used for humorous coping. It also appears that at times social media served as a distraction from difficult feelings that respondents were experiencing. A study by Lisitsa et al. (39) indicates the complicated impact of increased social media use during the pandemic, with data indicating both increased experiences of loneliness as well as increased support-seeking behavior; the determining factor appears to be whether the use was active or passive. The results from this study indicate that ability of social media to facilitate support-seeking was often used by the respondents in this survey. Similarly, other research has found that young people often go online to look for peers who are experiencing similar mental health difficulties and social media may be a way to facilitate this (31, 35, 41).

Discovery and “serendipitous finds” played an important role in the resources they did use. Many used Google search or the discovery pages on social media to find the resources they were using. Previous work also suggests that Google search is the natural first step for many young people seeking mental health support online with mental health literacy influencing which resources are selected (35). Similarly, respondents in this study indicated that many of them weren't aware of the formal online resources available to support them. Recommendations from peers also played an important role as did prior awareness of specific resources. The role of advertising and awareness campaigns on social media and in schools and colleges with this age group should not be discounted. The popularity of various influencers on social media also suggests that it might be worth partnering with certain influencers or making use of common hashtags to help gain visibility. It is worth considering how can we use this knowledge to direct young people to formal online resources, applying more guidance and structure to the discovery process. It has been widely recognized that whilst COVID-19 continues to be a threat to normal day-to-day living, a collaborative approach between educational institutions, community organizations, the health system, and parents is needed to ensure the mental well-being of young people (2).

Apps used for communication such as WhatsApp and Messenger were used by the majority (71.0%, 279/393) of participants to support their mental health. From the onset of the worldwide restrictions, there have been concerns about the effects that loneliness would have on young people's mental health (46). The ability to have real-life social contacts was no longer possible and so applications that could facilitate communication were heavily used. Orben et al. (3) note that although adolescence and young adulthood is an important period for social development, digital technologies offer the means to mitigate some of the negative effects associated with isolation from peers.

The COVID-19 pandemic presents significant threats to the mental health of young people, the opportunity exists to leverage digital health tools and resources to mitigate these effects (3, 47). The results from the qualitative questions in this survey highlight the factors that contribute to a “good” formal online resource and what is currently missing from the online resources they are using. These include personalization, opportunities for personal connection, accessibility, having meaningful, relevant choices available, visibility, and good design and layout. The design and delivery of appropriate online support services has become a necessity and is essential to ensure the ongoing support of young people's mental health (48).

## Strengths and Limitations

This study is the first to our knowledge to concentrate on the various digital tools used by young people to support their mental health during lockdown in Ireland. A strength of the study is that both quantitative and qualitative data was collected. The qualitative methods provided insights into the use of technology in context. This study has some limitations that warrant discussion. As this was an exploratory study, a cross-sectional study design was selected and therefore these results cannot speak to the continued use of digital tools at later points in the pandemic or how use changed over time. There are also limits to the generalizability of the findings of this survey as majority of the respondents were female, high school students. Participants were recruited from a limited number of online platforms (Facebook, Instagram, Twitter and SnapChat) limiting participation to young people who use these platforms. Finally, the survey was not piloted with young people to determine its acceptability; commentary and input from young people would be prioritized in future versions on this survey.

## Conclusion

COVID-19 and the associated lockdown measures has had significant implications for young people's health. It is evident that during the initial lockdown, young people used various online resources and digital tools to support their mental health and engage in various coping strategies. A collaborative but creative approach is needed to ensure young people are receiving the support they need; this requires leveraging the informal resources young people are already using to ensure they receive the formal support they require.

## Data Availability Statement

The raw data supporting the conclusions of this article will be made available by the authors, without undue reservation.

## Ethics Statement

The studies involving human participants were reviewed and approved by Ethics approval for this research was provided by the University College Dublin Office of Research Ethics (LS-20-23-Pretorius-Coyle). The patients/participants provided their written informed consent to participate in this study. Written informed consent was obtained from the individual(s) for the publication of any potentially identifiable images or data included in this article.

## Author Contributions

CP and DC formulated the survey questions. CP conducted the analysis. DC checked the analysis. CP wrote the manuscript whilst DC edited the manuscript.

## Funding

This publication has emanated from research conducted with the financial support of Science Foundation Ireland under Grant Number (12/RC/2289_P2). For the purpose of Open Access, the author has applied a CC BY public copyright license to any author accepted manuscript version arising from this submission.

## Conflict of Interest

The authors declare that the research was conducted in the absence of any commercial or financial relationships that could be construed as a potential conflict of interest.

## Publisher's Note

All claims expressed in this article are solely those of the authors and do not necessarily represent those of their affiliated organizations, or those of the publisher, the editors and the reviewers. Any product that may be evaluated in this article, or claim that may be made by its manufacturer, is not guaranteed or endorsed by the publisher.
