# Supplementary material for: Young People's Use of Digital Tools to Support Their Mental Health During Covid-19 Restrictions
Source: Front Digit Health. 2021 Dec 1;3:763876. doi: 10.3389/fdgth.2021.763876 (PMC8671300; doi:10.3389/fdgth.2021.763876)
Supplement: Supplementary file 1 [file Table_1.DOCX]

Supplementary Material

# Survey Questions

1. Demographic Information:

| Male |  | Female |  | Transgender |  | Other |  | Prefer not to say |  |
| --- | --- | --- | --- | --- | --- | --- | --- | --- | --- |

1. What is your age:

| 18 |  |
| --- | --- |
| 19 |  |
| 20 |  |
| 21 |  |
| 22 |  |
| 23 |  |
| 24 |  |
| 25 |  |

1. Please indicate the county in which you live:

| Carlow |  |
| --- | --- |
| Cavan |  |
| Clare |  |
| Cork |  |
| Donegal |  |
| Dublin |  |
| Galway |  |
| Kerry |  |
| Kildare |  |
| Kilkenny |  |
| Laois |  |
| Leitrim |  |
| Limerick |  |
| Longford |  |
| Louth |  |
| Mayo |  |
| Meath |  |
| Monaghan |  |
| Offaly |  |
| Roscommon |  |
| Sligo |  |
| Tipperary |  |
| Waterford |  |
| Westmeath |  |
| Wexford |  |
| Wicklow |  |

| City / Town |  |
| --- | --- |
| Rural area |  |

1. What is your current level of education?

| Fifth year |  |
| --- | --- |
| Sixth year |  |
| PLC Course |  |
| Undergraduate |  |
| Postgraduate |  |
| PhD |  |
| Apprenticeship |  |
| Other: |  |

We know this is a difficult time for many young people, social distancing can be difficult and

lonely. We’re interested in learning how you are using the Internet and online resources to

support your mental health during this time.

5. Which online resources are you using to support your mental health whilst practicing social distancing:

| A. | Yes / No | Website |
| --- | --- | --- |

If yes, which websites are you using to support your mental health: ___________________________________________

| B. | Yes / No | Social media |
| --- | --- | --- |

If yes, which platforms:

| Instagram | Facebook | Snapchat | Twitter | TikTok | YouTube | Tublr | LinkedIn |
| --- | --- | --- | --- | --- | --- | --- | --- |

(for each platform selected above, the following question will be asked)

Please name the accounts/channels you use on this platform for the purpose of supporting your mental health: ________________________

| C. | Yes / No | Discussion board |
| --- | --- | --- |

If yes, which ones:

| Reddit | Boards.ie | Other: |
| --- | --- | --- |

| D | Yes / No | Charity / health service websites |
| --- | --- | --- |

If yes, which ones:

| HSE | Jigsaw | SpunOut | Samaritans | Pieta House | Turn2Me | A Lust for Life | Aware | Other: |
| --- | --- | --- | --- | --- | --- | --- | --- | --- |

| E | Yes / No | An App |
| --- | --- | --- |

If yes, which ones

| Silvercloud | Headspace | Calm | Other: |
| --- | --- | --- | --- |

| F | Yes / No | Communication apps |
| --- | --- | --- |

If yes, which ones:

| WhatsApp | WeChat | Facebook messenger | Skype | Google hangouts | Other: |
| --- | --- | --- | --- | --- | --- |

| G | Yes / No | Professional counselling/therapy services offered online |
| --- | --- | --- |

If yes, please specify which services you’re using:

|  |
| --- |

| h | Yes / No | Other |
| --- | --- | --- |

Please specify: __________________________________________

6. How are you finding online mental health supports?

| A | Google search |
| --- | --- |
| B | Social media ads |
| C | Recommendations from friends/peers |
| D | Recommendations from school/ college |
| E | Recommendations from parents |
| F | Prior awareness of specific mental health supports |
| G | By following links on websites already used |
| H | Other: |

7. How would you describe your experience of looking online for mental health supports?

|  |
| --- |

8. What online resources are you using now that you already made use of before social distancing was implemented?

|  |
| --- |

9. What online resources are you using now that you **were not** using before social distancing was implemented?

|  |
| --- |

10. Would you continue to use these resources once social distancing has ended and why?

|  |
| --- |

11. For each online resource you selected yes for above, please indicate your level of satisfaction with this resource?

| Not sure | Very dissatisfied | dissatisfied | neutral | satisfied | Very satisfied |
| --- | --- | --- | --- | --- | --- |

12. What makes a good online resource?

|  |
| --- |

13. How important is it that the following things are incorporated into an online resource:

|  | Not important at all | Not important | Doesn’t matter | Important | Very important |
| --- | --- | --- | --- | --- | --- |
| Information presented through clear, easy to read text |  |  |  |  |  |
| Information presented in videos |  |  |  |  |  |
| Information presented in podcasts |  |  |  |  |  |
| Personal stories of other people who might have similar experiences |  |  |  |  |  |
| A way to connect to a health professional online |  |  |  |  |  |
| A way to connect to other people my age online |  |  |  |  |  |
| A search bar |  |  |  |  |  |
| A quiz of my symptoms to help me find what I’m looking for |  |  |  |  |  |
| A discussion board |  |  |  |  |  |
| Links to other online resources |  |  |  |  |  |
| Activities I can do right now to make me feel better |  |  |  |  |  |
| Activities I can do on an ongoing basis to help me feel better |  |  |  |  |  |

14. What do you think is missing from the online resources you’re currently using to support your mental health?

|  |
| --- |

**Support Services**

***The services listed below may be of benefit if you are looking for information or support.***

**Samaritans:** 
Phone: 116 123 (Freephone, Republic of Ireland)

Email: jo@samaritans.org

*24 hours a day, 365 days a year*

**If you are in crisis:**

- Contact your local doctor. To find yours, visit **www.icgp.ie/go/find_a_gp**
- Contact the Accident and Emergency Department of your nearest general hospital
- Emergency services can be contacted at any time by dialling 999 or 112.

**Mental health information and support services:**

**www.spunout.ie** - offers comprehensive information on all aspects of mental health as well as other topics relevant to young people. An anonymous text line is also available to you to talk to a trained volunteer.

**www.turn2me.org**  - provides a variety of support options, including online counselling, online support groups and live chat.

**www.yourmentalhealth.ie –**provides information on mental health and support services near you.

**www.bodywhys.ie** – offers online support for eating disorder issues, including an online group counselling service.

**www.drugs.ie** – offers drug and alcohol information and support including a ‘live helper’ service that lets you chat to a staff member online.

# Tables

Table 7. Motivation for continued use of online resources post-lockdown

| Theme | Sub-codes | Description | Sample quote |
| --- | --- | --- | --- |
| Overall beneficial experience | Discovery | Yes. Discovered a resource, tool or behavior facilitated by an online resource, that’s always been available but would never have tried if it wasn’t for lockdown. | “Yes. I didnt realise how helpful spunout really was. Ive never given it a chance before. Desperate times made me!” |
|  | Beneficial experience | They found the technological resources they’ve been using helpful | “Yes because I find them reassuring and they help me feel less alone.” |
|  | Sense of community | A sense of belonging, community. Others they can identify with. | “Yes, it gives a good insight into other young peoples’ life experiences, and can make you feel less alone” |
|  | Informative | Have found certain online resources beneficial because of they are informative | “Yes, they have valuable information on how to deal with mental health issues” |
|  | Physical benefits | Physical wellbeing - engaging in physical activities and meditative practices that have a positive impact on both mind and body and encouraged to keep using these | “Yes Because I have found that working out has really uplifted my mood” |
| Addressing an ongoing need | Useful because it’s calming | The online resource helps to manage feelings of stress and anxiety | “Here relaxing reduces stress” |
|  | Resources previously used | May have identified or been managing mental health difficulties before pandemic and were making use of resources, which are resources they used during lockdown and will continue to use after lockdown. | “In general my main struggles are to do with isolation & loneliness, so it hasn’t really changed since lockdown hence, I’ve just continued using them.” |
|  | Needing continued mental health support | Respondent acknowledges that even after lockdown their mental health will need to be addressed and supported. | “I’ll probably need to get some sort of psychological help after all of this and my mind has wandered under the stress of college and the current pandemic” |
|  | Accessibility | Technological resources offer increased accessibility | “Yes I think so, because it's handy to have something that I can access remotely online from the comfort of my own home” |
| Online resources used as a substitute for offline experiences | Preference for in-person-mental health professional | There is a preference for in-person support from a mental health professional. This can be a counsellor, psychologist, therapist, who is either private, state or college based. | “I'll definitely go back to CBT in person - more privacy than at home and I'm not worried about people over hearing me. It does work very well though.” |
|  | Preference for in-person-social group | Online technologies have facilitated online social relationships, they’re a current substitute for offline experiences but this will not continue after lockdown | “I would, but in a much more limited capacity. I use them as a substitute for talking to friends, and I look forward to meeting them again in person. I can feel lonely and frustrated when I can't see anyone but my family” |
| Unhelpful experience | Not useful | The online resource being used wasn’t helpful and therefore won’t continue using them. | “No because it didn't help” |
|  | Not a specific resource | There are certain resources that won’t be used after lockdown, but this doesn’t mean all online resources | “I'm not sure if I will use SilverCloud, mainly because I don't find myself using it as much as others” |

Table 8 What makes a good online resource

| Theme | Sub-codes | Sample quote |
| --- | --- | --- |
| Interpersonal connection | Preference for personal contact | “Ability to talk to another person (not just articles, videos, etc)” |
|  | Connecting with. Mental Health professional | “Professional contacts is a must. It is sadly sometimes a race against time and therefore a resource to talk straight away is needed.” |
|  | Connecting with peers | “A neutral ground where you can talk to others that feel the same.” |
|  | Empathic experience | “Ability to disclose all information and feel listened to and heard rather than just receiving a common and quick remedy” |
| Content | Advice, activities, and skills | “through giving me the coping skills to deal with my own emotions.” |
|  | Information | “Accurate information that’s easy to understand” |
|  | Linking to other resources | “Links to other related topics; helps find what you are looking for” |
|  | Personal stories | “Personal accounts and witness; sometimes easier to identify with then neutral information” |
|  | Interactivity features | “Interactive options - polls, comments, short reminders that the resource is for the user.” |
| Accessibility | Accessibility | “Accessible (free is best, not in the position to pay for therapy/counselling)” |
|  | Anonymity | “Anonymous option” |
|  | Confidentiality and privacy | “Confidentiality” |
| Functionality | Medium of communication | “Even type or text out as I know plenty if young people would have issues with talking to someone, even over the phone. Sometimes texting is easier.” |
|  | Tailoring | “Contact with a person that has tailored advice. All is fine for the usual “it will be okay”, “it’s normal to feel like this sometimes” gets monotonous and useless.” |
|  | Design and layout | “Simple, clear layout. Chances are someone visiting the resource may already be overwhelmed.” |

Table 9 What’s currently missing from online resources

| **Theme** | **Sub-codes** | **Quote** |
| --- | --- | --- |
| Accessibility | Content for marginalized or specific groups | “I would say supports aimed specifically at LGBT+ issues” |
|  | Free or low cost | “A resource or app that is completely free, I find the applications like headspace, calm and mindfulness in 8 weeks all cost money which alot of young adults cant give/ afford.” |
|  | Less pathological approach | “I find some resources present information from a pathological standpoint e.g. mental health disorders but often all I need is advice for having a few bad days or a stressful few weeks.” |
| Interpersonal connections | Connecting with mental health professional | “A means through which I can access a professional mental health worker” |
|  | Connecting with peers | “A way to connect with other people who have had similar experiences” |
|  | Discussion board | “Discussion boards perhaps would be helpful where we could post an anonymous question and discuss it with other young people” |
|  | Interpersonal connection | “I feel like when it is online there is a lack of personal contact. As if it's impossible to truly voice your opinions and get immediate feedback specific to you as an individual” |
| Tailoring | Personalisation | “A lot of advice is very generic and doesn't fit a lot of people. We need to hear from a range of different people to dinf something instead of just hearing generalised tips and tricks” |
|  | Appropriate, tailored guidance | “Advice of substance. Something actually helpful with regards to study and being apart from friends, rather than being told to keep my head down or go for a walk.” |
| Meaningful, relevant choices | Appropriate, relatable information | “More indepth information for someone like me who wants to understand more about why and how I'm feeling. Just because we're young doesn't mean the information can't be detailed. Also, resources addressing what we're actually going through. Things addressing the Leaving Cert or college through the CAO directly will make it easier to relate to, and more effective for vulnerable Irish students.” |
|  | Varied content | “Diversified modes of presentation eg not only videos, but also blog posts, podcasts etc” |
|  | Actionable steps and skills | “Activities to help with mental health struggles, whether ongoing or current” |
|  | Chat | “anonymous chat room/ somewhere to text for help instead of ringing” |
| Visibility | Advertising | “Current resources seem to be passively giving information and allowing people to find it instead of playing an active role in putting forward their information and actively helping you as an individual as opposed to a generalised group of people with all different issues” |
| Design and Layout | Design and layout | “A friendly user interface that looks inviting and allows you to feel like asking for help is totally fine” |
